# A manganese porphyrin-based T1 contrast agent for cellular MR imaging of human embryonic stem cells

**DOI:** 10.1038/s41598-018-30661-w

**Published:** 2018-08-14

**Authors:** Andrei Venter, Daniel A. Szulc, Sadi Loai, Tameshwar Ganesh, Inga E. Haedicke, Hai-Ling Margaret Cheng

**Affiliations:** 1 Institute of Biomaterials & Biomedical Engineering, University of Toronto, Toronto, Canada; 2 Translational Biology & Engineering Program, Ted Rogers Centre for Heart Research, Toronto, Canada; 3 Leslie Dan Faculty of Pharmacy, University of Toronto, Toronto, Canada; 4 The Edward S. Rogers Sr. Department of Electrical and Computer Engineering, University of Toronto, Toronto, Canada; 5 Heart & Stroke/Richard Lewar Centre of Excellence for Cardiovascular Research, Toronto, Canada; 6 Ontario Institute for Regenerative Medicine, Toronto, Canada

## Abstract

MRI for non-invasive cell tracking is recognized for enabling pre-clinical research on stem cell therapy. Yet, adoption of cellular imaging in stem cell research has been restricted to sites with experience in MR contrast agent synthesis and to small animal models that do not require scaled-up synthesis. In this study, we demonstrate the use of a gadolinium-free T1 contrast agent for tracking human embryonic stem cells. The agent, MnPNH_2_, is an easily synthesized manganese porphyrin that can be scaled for large cell numbers. MRI was performed on a 3 T clinical scanner. Cell pellets labeled at different MnPNH_2_ concentrations for 24 hours demonstrated a decrease in T1 relaxation time of nearly two-fold (*P* < 0.05), and cellular contrast was maintained for 24 hours (*P* < 0.05). Cell viability (Trypan blue) and differentiation (embryoid body formation) were unaffected. Cell uptake of Mn on inductively coupled plasma atomic emission spectroscopy corroborated MRI findings, and fluorescence microscopy revealed the agent localized mainly in cell-cell boundaries and cell nuclei. Labeled cells transplanted in rats demonstrated the superior sensitivity of MnPNH_2_ for *in-vivo* cell tracking.

## Introduction

Human stem cells have the unique potential of renewing themselves and differentiating into tissue-specific cells with specialized function, thus representing a clinically relevant cell source in regenerative medicine^1,2^. Embryonic stem cells (ESCs), derived from the inner cell mass of the blastocyst, are favored for their potential to treat a variety of diseases and injuries, including heart disease, stroke, diabetes, and bone and cartilage deterioration^3^. However, despite continued advances in stem cell-based regeneration strategies, a number of critical barriers related to cell delivery and tracking must still be overcome. There is an urgent need for novel methods to non-invasively track ESCs *in vivo*. Currently, we are completely blind as to the fate of cells post-transplantation and must await histology to confirm successful engraftment. The ability to label and visualize ESCs *in vivo* would help ensure their appropriate distribution within the tissue during initial delivery, and it would allow assessment of graft cell death and function over time (e.g. informing the need for additional cell injections and/or modulated immunosuppression).

Magnetic resonance imaging (MRI) is a sensitive and non-irradiative approach for non-invasive cell tracking *in vivo*^4^. Over the years, various applications have been reported, the majority of these utilizing iron-oxide nanoparticles for cell labeling^5^. However, the non-specificity of a dark signal, which can arise from the iron-labeled cells or from endogenous T2 sources (e.g. blood clots) or from macrophages that have ingested iron released from dying cells^6^, has prompted efforts to use bright contrast instead for cell labeling. To this end, both gadolinium and manganese have been explored as potential T1 agents for cell labeling and tracking^7,8^. While toxicity issues exist with gadolinium^9^, manganese (Mn) is naturally found in the body and any excess at low concentrations can be eliminated safely. Therefore, while manganese should not be used in its ionic form (e.g. MnCl_2_), when bound tightly, the Mn ion can exert its T1 effects without posing risks to the body.

In this study, we propose a Mn-based contrast agent for cell labeling, one that is easy to synthesize and has high thermodynamic stability^10^, both important factors for eventual clinical translation. The proposed agent, hereafter termed MnPNH_2_, is a monomeric Mn porphyrin modified with a single amine group for enhanced cell uptake. We have previously investigated the relaxation properties of this agent in solution and found it to be far superior to gadolinium agents used clinically, with *r*1 = 9.33 mM^−1^s^−1^ and *r*2 = 12 mM^−1^s^−1 ^^11^. Here, we investigate the efficacy of MnPNH_2_ for labeling human ESCs and confirm the absence of adverse effects on cell viability, colony formation, suspended cell aggregation behavior, and differentiation. We also provide the first characterization of the agent’s subcellular distribution and efficacy *in vivo*. Our overall goal is to advance MRI contrast agents that are not only safe and provide sensitive cell detection *in vivo* but also are easy to synthesize for scalability, to enable studies in larger animal models and eventually patients receiving stem cell treatment.

## Materials and Methods

### Chemicals for Synthesis

All reagents and deuterated solvents used for synthesis were of reagent grade or better and were used without further purification unless stated otherwise. Starting materials, reagents and deuterated solvents were purchased from Sigma Aldrich, and all other solvents were purchased from Caledon Laboratories. The PNH_2_ precursor, 5-(4-aminophenyl)-10, 15, 20-(tri-4-sulfonatophenyl)porphyrin triammonium, was purchased from PorphyChem. All reactions were carried out under argon. Thin layer chromatography was carried out on pre-coated aluminum plates of Silica Gel 60 F254 from Merck. Column chromatography was performed using Caledon Silica Gel 60. Dialysis was performed with Biotech CE dialysis tubing (MWCO 100–500 Da). Cation exchange was performed using an Aberlite IR120 H resin. All spectroscopic data for structural characterizations were obtained using the research facilities in the Department of Chemistry. NMR spectra were recorded on a Brucker-500 MHz. UV-visible spectra were recorded on an Agilent 8453 system. HPLC spectra were recorded on a PerkinElmer SERIES 200 system. FAA spectra were recorded on a PerkinElmer AAnalyst 100 system. Mass spectroscopy was carried out on a Agilent 6538 Q-TOF system.

### Synthesis of 5-(4-aminophenyl)-10,15,20-tris(4-sulfonatophenyl) porphyrinato manganese (III), MnPNH_2_

The proposed contrast agent is a monomeric manganese tetraphenyl porphyrin with three sulfonate groups to afford water solubility and one amine group for improved cell permeability relative to the well-known manganese complex of 5, 10, 15, 20-tetra(sulfonatophenyl) porphyrin.

**Figure 1:**
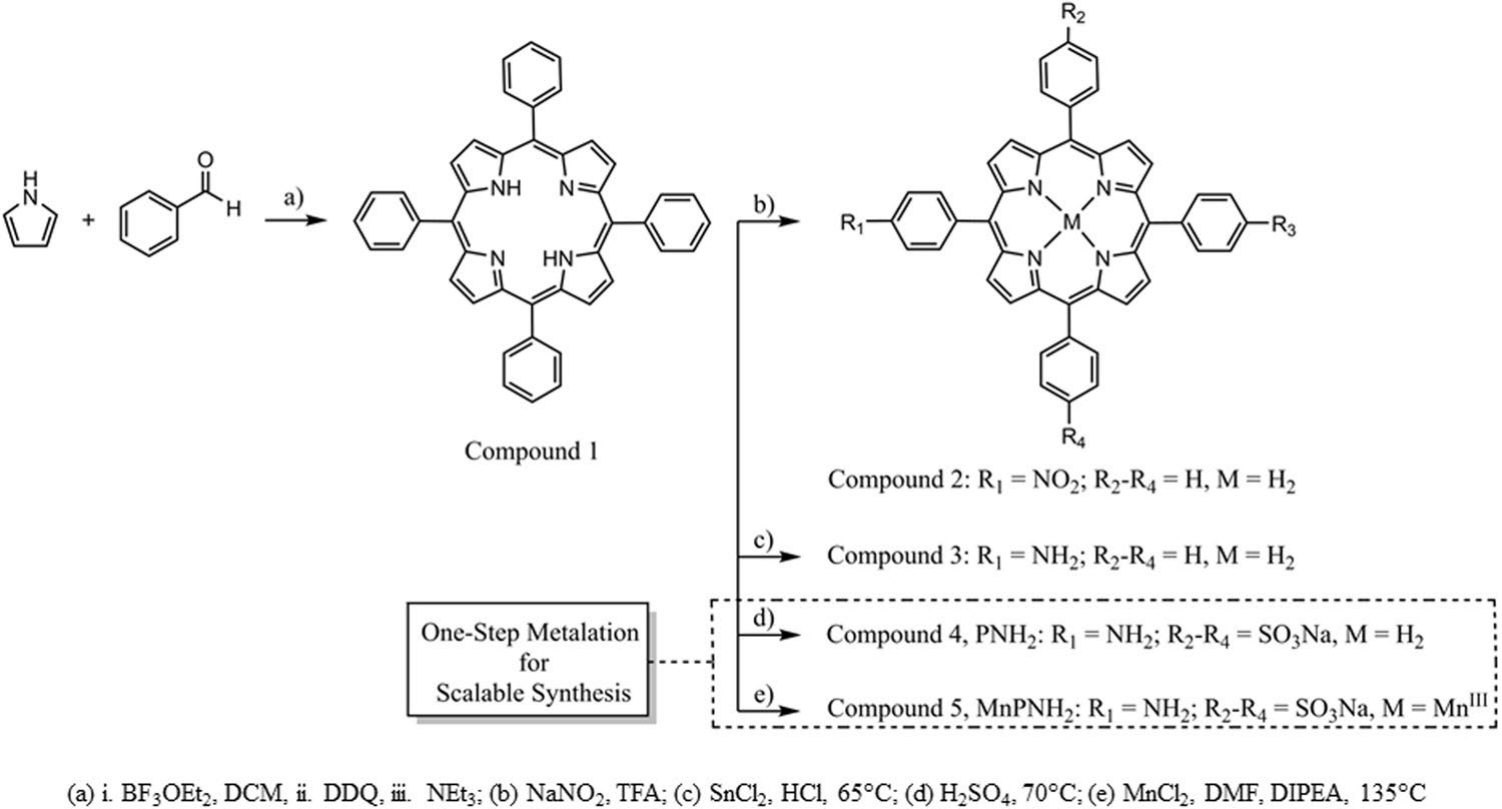
Schematic of chemical synthesis. The synthesis of MnPNH_2_ from simple starting materials and the one-step metalation from the commercial precursor PNH_2_ is shown.

The contrast agent, MnPNH_2_, was synthesized according to previously described procedures^12–14^; the full and scalable synthetic routes are shown in Fig. 1. The first step involved a condensation reaction between pyrrole and benzaldehyde carried out in dichloromethane with boron trifluoride etherate as the acid catalyst followed by oxidation with DDQ to provide compound 1, tetraphenyl porphyrin in 40% yield^12^. Subsequent nitration of the para-position of the phenyl ring with sodium nitrite in trifluoroacetic acid provided a mixture of compound 2 and dinitroporphyrins^13^. This mixture was carried through to the hydrochloric acid-tin (II) chloride catalyzed reduction of the nitro groups to provide aminophenyl porphyrin, compound 3^13^ in 56% yield. Finally, compound 3 was heated in concentrated sulfuric acid to provide 84% of the desired compound 4, PNH_2_^14^. Mn was then inserted into compound 4 by metalation with MnCl_2_ in dimethylformamide and N,N-Diisopropylethylamine with heat for 3 hours, to produce the final product, compound 5, MnPNH_2_. This final step was also repeated with the purchased PNH_2_, compound 4. The structures of compounds 1 and 3 were confirmed by ^1^H NMR. Compound 4, PNH_2_, was characterized by ^1^H NMR, mass spectrometry, HPLC and UV-Visible spectroscopy matching the literature. Compound 5, MnPNH_2_, synthesized from both the purchased and in-house produced compound 4, was characterized by mass spectrometry, UV-Visible spectroscopy, HPLC, and FAA spectrometry matching literature.

### Human Embryonic Stem Cell Line and Cell Culture

Human ESCs from the line ESI–017 (ESIBio, SKU: ES-700) were cultured in sterile conditions on tissue culture plates coated with Corning™ Matrigel™ Membrane Matrix (Fisher Scientific Catalog No.08-774-552) and kept in an incubator at 37 °C and 5% CO_2_. Cells were grown in colonies, maintained in mTeSR ™1 (STEMCELL Technologies Catalog # 85850), and passaged using enzyme-free dissociation to prevent differentiation and allow cells to remain in small colonies using Gentle Cell Dissociation Reagent (STEMCELL Technologies Catalog #07174) and mechanical cell scraper separation.

### Cell Labeling Studies

Stock solution of MnPNH_2_ at 10 mM dissolved in sterile distilled water under sterile conditions was created to label cells without significantly changing the volume of media. This stock solution was then heated at a low boil for 5 minutes to further ensure sterility. The contrast agent was added directly from the stock solution into the well containing cells that were to be labeled.

At the end of the cell labeling interval, the cell media was removed and cells were rinsed with room temperature DPBS (Thermofisher Catalog # 21600010) three times with gentle swirling of each rinse to ensure that most of the contrast agent not taken up by cells was washed off. The stem cells were then dissociated using a gentle cell dissociation protocol and then fully removed with a cell scraper. Cells were then resuspended in PBS and centrifuged at 300 g three times for five minutes each time to ensure all extracellular contrast agent was fully removed. The cells were then transferred into 115 × 5 mm Wintrobe sedimentation tubes (Kimble Chase, Vinelad, NJ), topped with mTeSR media, and transported on ice to the MRI scanner.

A range of contrast agent concentrations for cell labeling (0.1–0.5 mM) and two different labeling intervals (2 and 24 hours) were tested. The retention of contrast in labeled cells was also investigated up to 4 days post-labeling.

### *In-vitro* MRI

Immediately after pelleting, the cell pellet-containing tubes were taken to MRI and placed in a custom-made ULTEM™ resin holder. Imaging was performed on a clinical scanner (Achieva 3.0 T TX, Philips Medical Systems) using a 32-channel head coil. T1 mapping was performed using inversion recovery turbo spin echo: TR = 3000 ms, TE = 18.5 ms, 5 cm field-of-view, 3 mm slices, 0.5 × 0.5 mm in-plane resolution, and TI = [50, 100, 250, 500, 750, 1000, 1250, 1500, 2000 and 2500] ms. After image acquisition, the data was analyzed on a 3-mm deep cylindrical volume within each cell pellet. T1 values were calculated pixel-by-pixel (~50 pixels per vial) using in-house software developed in Matlab (ver. 8.1) following the method of ref.^15^.

### Quantification of Intracellular Manganese Content

To quantify manganese content on a per-cell basis, 0.2 mL aliquots of the previously imaged cell pellets were digested by the addition of one millilitre of 70% ultrapure analytical grade HCl and sonication at 40 °C for 30 minutes. The solution was then diluted to a final volume of 6 mL with ultrapure water. The final solution was run through a 0.22-µm filter to remove residual large protein, thus leaving a solution containing the MnPNH_2_ that was taken up by cells. These samples were then run on an inductively coupled plasma atomic emission spectrometer ICP-AES (Optima 7300 DV ICP AES). ICP-AES passes this solution through a plasma flame to excite the manganese, which then emits a signal at 293 nm that is converted into parts-per-billion (ppb) of Mn in the solution. This value was then divided by the number of cells to give an approximation of the amount of Mn in one cell.

### Cell Viability

To assess the effect on MnPNH_2_ on cell viability, human ESCs were grown in 6-well plates until colonies reached 60% confluency. Wells were labeled with MnPNH_2_ for 24 hours at 0, 0.1, 0.2, 0.3, 0.4, and 0.5 mM. Once labeling was complete, the contrast agent-containing media was aspirated, and cells were rinsed three times with room temperature PBS to eliminate residual extracellular contrast agent. Since human ESCs grow in colonies, complete dissociation into single cells was necessary to perform the trypan blue assay. The cells were then removed from the wells and suspended in 3 mL of PBS in 15-mL tubes for counting. Aliquots of 1 mL volume from each sample were then automatically mixed with trypan blue and counted on a Vi-Cell XR Cell Viability Analyzer (Beckman Coulter) with default image gating. Fifty separate images were taken and counted for viability. This trial was repeated three separate times to ensure statistical relevance.

### Stem Cell Differentiation into Embryoid Bodies

The essence of using stem cells for therapy is their ability to differentiate into different cell types. Therefore, any contrast agent employed for cell labeling and tracking cannot adversely affect differentiation potential. To confirm that labeled human ESCs maintained their innate ability to differentiate, four wells of a six-well plate with roughly 60% confluent human ESC colonies were labeled with MnPNH_2_ for 24 hours, with two wells at 0.2 mM and two other at 0.5 mM. After labeling, the contrast agent-containing media was aspirated and cells were rinsed three times with room temperature PBS to eliminate residual extracellular contrast agent. The cells were then removed and plated into 12-well untreated, uncoated plates for suspension culture. Each original well was split into 6 of the 12-well plate wells with 2 mL of complete mTeSR media and incubated on a shaker spinning at 60 rpm and left for 5 days to assist aggregation.

Media was subsequently changed every four to five days with care not to disturb the embryoid bodies until day 14 for imaging. To prepare for imaging, the embryoid bodies were gently rinsed three times with PBS, fixed with 4% paraformaldehyde for 10 minutes at room temperature, and labeled with DAPI nuclear stain (Thermofisher Catalog # D1306).

### Subcellular Contrast Agent Distribution

The subcellular distribution of a contrast agent determines the degree of contrast change obtained on imaging. To gain insight into where MnPNH_2_ distributes intracellularly, we employed the apo-version, PNH_2_, without the fluorescence-quenching Mn ion. This apo-version is a fluorescent compound with peak absorption around 415 nm and emission around 650 nm^16^. Human ESCs colonies were grown on glass cover slips to 60% confluency and labeled with mTeSR media containing 0.5 mM of MnPNH_2_ for 24 h. After labeling, cells were rinsed three times with room temperature PBS and fixed with 4% paraformaldehyde at room temperature for 10 minutes. The cover slips were then mounted and imaged on a Leica DMi8 Inverted Microscope using the excitation filter of a DAPI filter cube (350/50 nm) and a DSRED emission filter (605/75 nm). A control sample of unlabeled human ESCs was also prepared as above.

### *In-vivo* Rat Study

This study was approved by the Lab Animal Services of the Hospital for Sick Children (protocol #41181), and all procedures were conducted in accordance with the Canadian Council on Animal Care. Cells were labeled for 24 hours with MnPNH_2_ at 0.22 mM, which is approximately less than half the maximum concentration tested *in vitro*. Immediately after labeling, cells were collected, suspended in mTeSR media in Falcon tubes, and transported to the MR scanner. Female adult Sprague Dawley rats (*N = *2) (Charles River Laboratories) weighing 200 g were anesthetized on 3% isoflurane (Forene, Abbott Labs, Baar, Switzerland) in pure oxygen (2 L/min flow rate). Approximately 10 million cells in 0.1 mL media was injected subcutaneously on the dorsal side close to the midline. One rat received an injection of labeled cells, and the other received an injection of unlabeled cells (control). Both rats were also given 0.2 mL saline injections as negative controls. Each rat was then placed prone in an 8-channel wrist coil, lying on a water blanket (HTP-1500, Adroit Medical Systems, Loudon, TN) set at 38 °C to maintain core body temperature. A maintenance dose of 2% isoflurane in pure oxygen was applied throughout imaging. Twenty 1-mm thick sagittal images slices were positioned centered at midline. A 2D T1-weighted spin echo sequence with fat suppression was acquired: repetition time (TR) = 724 ms, echo time (TE) = 13.6 ms, number of signal averaging (NSA) = 3, field of view (FOV) = 100 mm, and 0.6 × 0.6 mm in-plane resolution. To visualize the fluids in all injectate, a 2D T2-weighted turbo spin echo sequence was acquired with the same pixel resolution: TR = 4000 ms, TE = 75 ms, NSA = 2, echo train length = 16.

### Statistics

Differences in T1 relaxation times were determined using Analysis of Variance (ANOVA). Changes in T1 with incubation concentrations and labeling interval were analysed with two-way ANOVA, while changes in T1 with post-labeling interval were analysed with one-way ANOVA. Post-hoc analysis was based on the Tukey-Kramer test. Significant changes in cell viability were determined using a two-tailed Student’s t-test. Significance was reported at a *p*-value of 5%.

## Results

**Figure 2:**
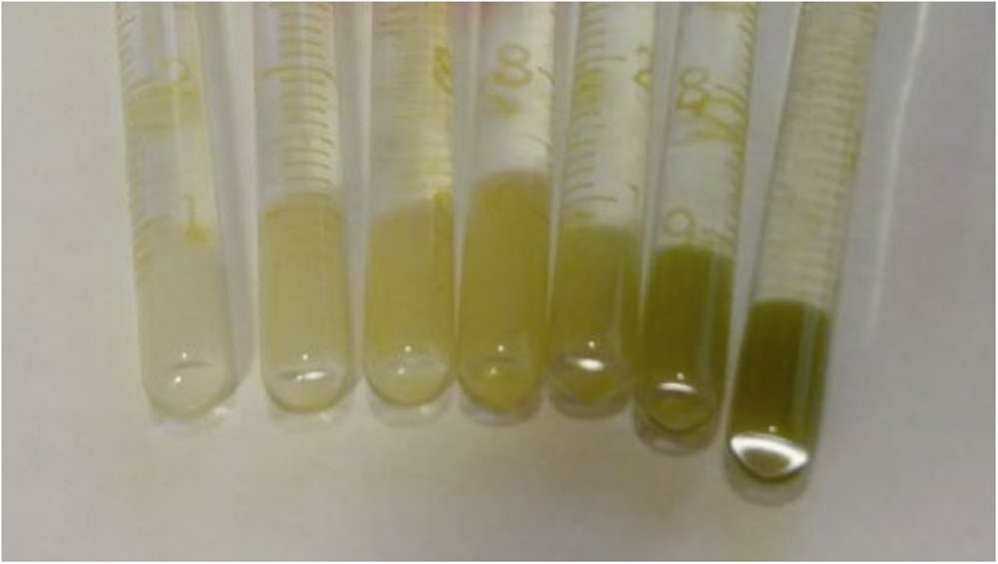
Labeled human embryonic stem cells prepared for MR imaging. Cell pellets in borosilicate glass tubes show characteristic dark green of the porphyrin contrast agent. A darker green corresponds to increased contrast uptake.

Figure 2 shows that the success of labeling cells with MnPNH_2_ can be qualitatively observed by the color of the cell pellets. A gradient of color from white (i.e. unlabeled cell pellets) to progressively darker green can be seen, correlating well with the MnPNH_2_ concentration used for labeling. The staining can be discerned even on a cellular level.

**Figure 3:**
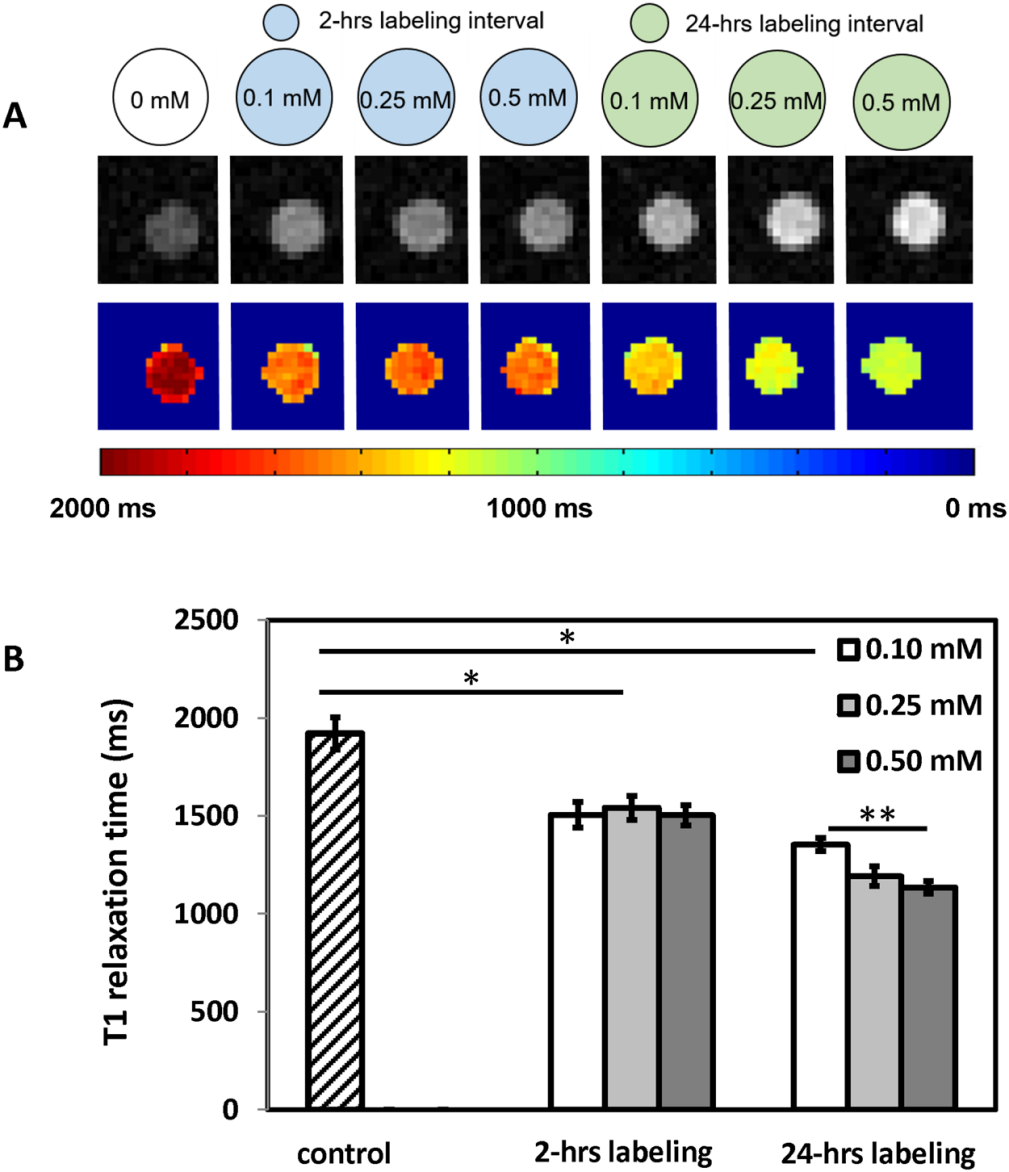
Impact of labeling conditions on reductions in T1 relaxation times (**A**) MRI of labeled human embryonic stem cells on 3-Tesla scanner. T1-weighted inversion-recovery images of cells labeled for different time intervals (2 and 24 hours) and various incubation concentrations (top row), and corresponding T1 map (bottom row). (**B**) Mean T1 relaxation times for unlabeled cells and human embryonic stem cells labeled under different conditions. Error bars represent standard deviation. ^*^Denotes significant difference (P < 0.05) between control and different labeling intervals. ^**^Denotes significant difference (P < 0.05) with contrast concentration within the same labeling interval.

**Figure 4:**
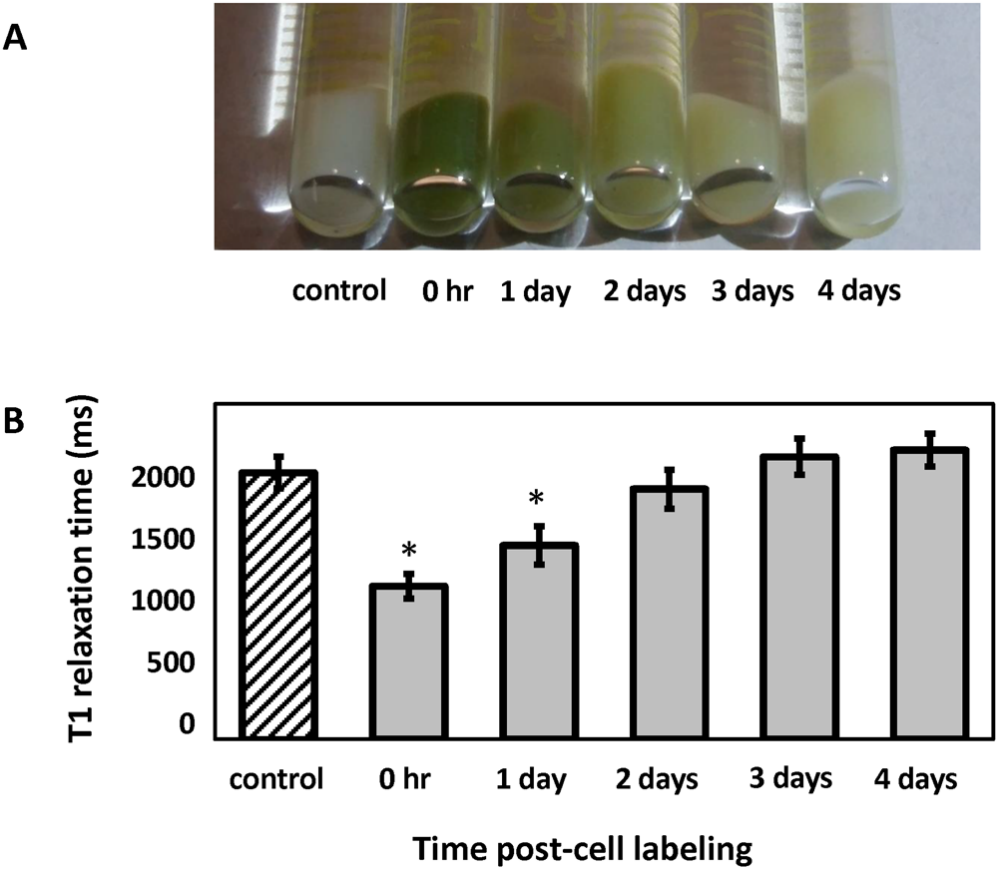
Retention of contrast in labeled human embryonic stem cells. (**A**) Cell pellets visibly lose the green color imparted by the porphyrin contrast agent with longer intervals post-labeling. (**B**) T1 relaxation times for unlabeled control cells and cells labeled at 0.5 mM MnPNH_2_ for 24 hours at different times post-labeling. T1 has returned to baseline levels by 48 hours. Error bars represent standard deviation. *Denotes significant difference (P < 0.05) between control and different post-labeling intervals.

The efficacy of MnPNH_2_ as a T1 contrast agent is shown in Fig. 3. Significant decreases in T1 relaxation times were achieved even for short labeling intervals and low incubation concentrations. The decreases in T1 in all labeled cells were significantly different from unlabeled controls (*P* < 0.05), and there was a significant difference between the two labeling intervals of 2 and 24 hours (*P* < 0.05). However, the anticipated dependence of T1 on incubation concentration was observed only for a labeling interval of 24 hours but not 2 hours. Table 1 summarizes intracellular Mn quantification from ICP-AES, which is seen to corroborate T1 relaxivity measurements. A retention study of cells labeled at 0.5 mM MnPNH_2_ for 24 hours revealed that substantial contrast remained within the first 24 hours post-labeling but thereafter decreased towards baseline levels, with insignificant T1 contrast after 2 days (Fig. 4).

**Table 1:** Manganese content in cell pellet digests as measured on ICP-AES.

Sample	Mn Concentration/pellet (ppb)	Mn Concentration/cell (mol)
Control	BDL	BDL
**2-hour labeling interval**		
0.10 mM	8.82	1.05e-11
0.25 mM	13.5	1.61e-11
0.50 mM	19.1	2.28e-11
**24-hour labeling interval**		
0.10 mM	12.4	1.47e-11
0.25 mM	18.0	2.14e-11
0.50 mM	46.9	5.59e-11

BDL: below detection level.

**Figure 5:**
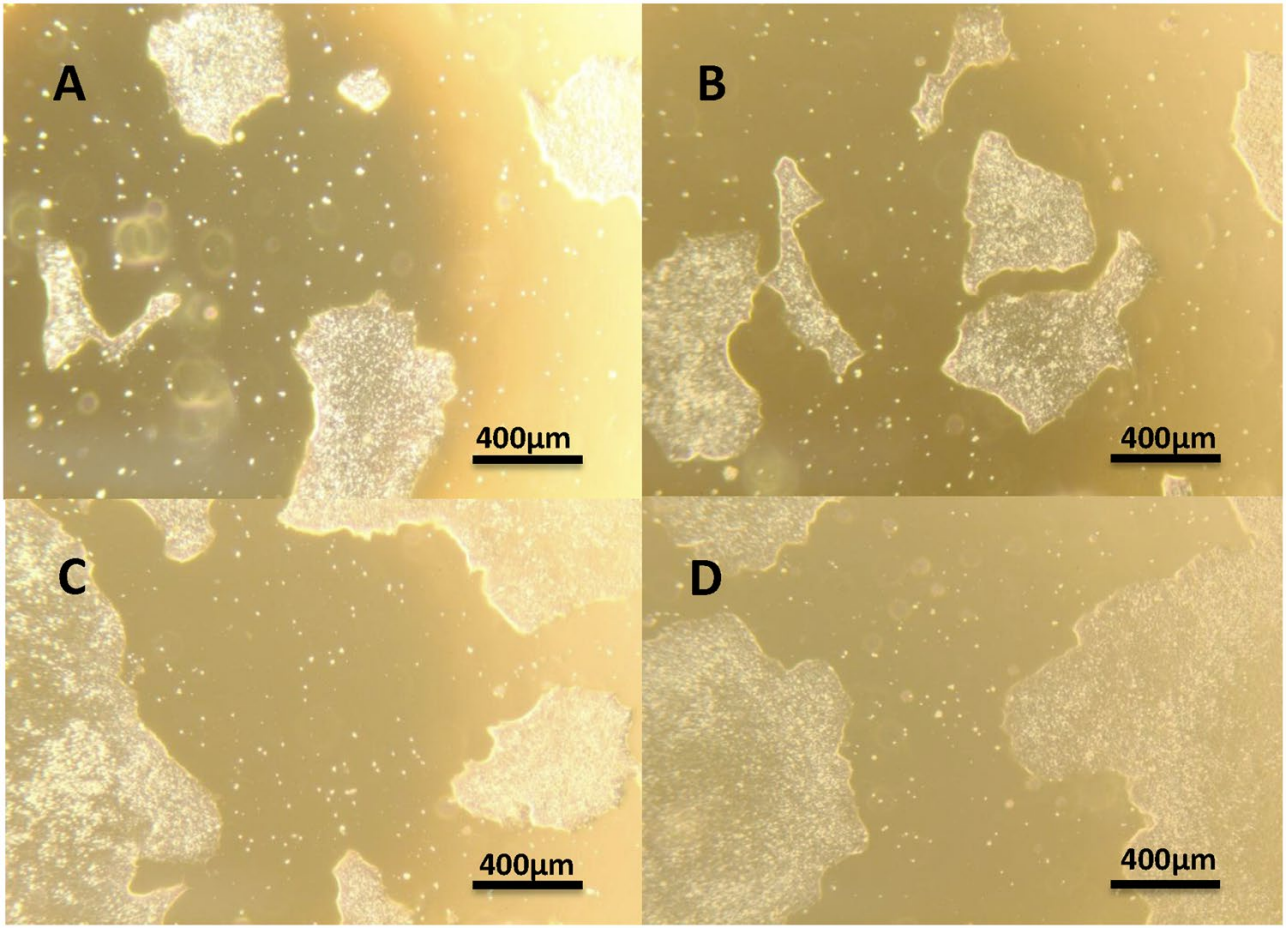
Phenotype of human embryonic stem cells on microscopy. Representative bright-field images showing human embryonic stem cell colonies before labeling and at 24, 48, and 72 hours after labeling at 0.5 mM MnPNH_2_ for 24 hours. Cell morphology and colony shape are unchanged (4× magnification).

**Figure 6:**
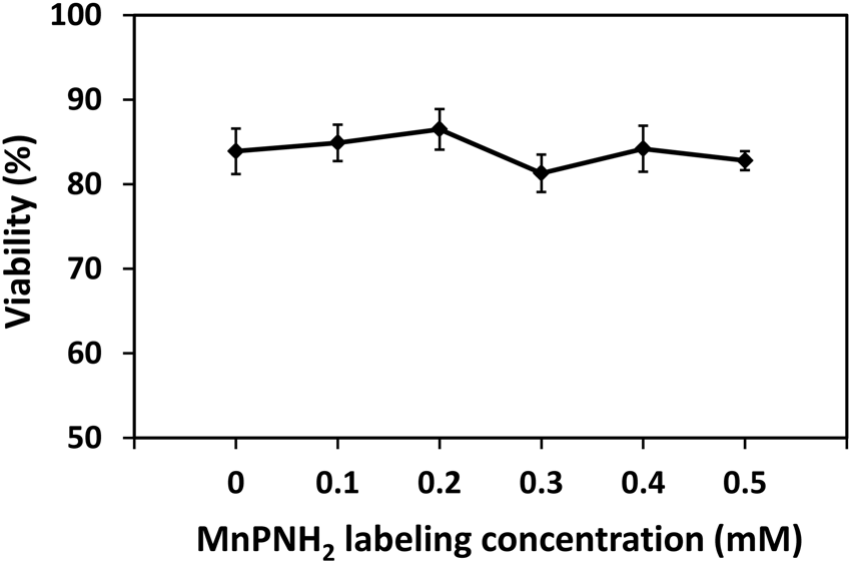
Cell viability. Viability assessed on trypan blue assay for different incubation concentrations and 24-hour labeling. Shown are mean values and standard deviation (*N* = 3).

**Figure 7:**
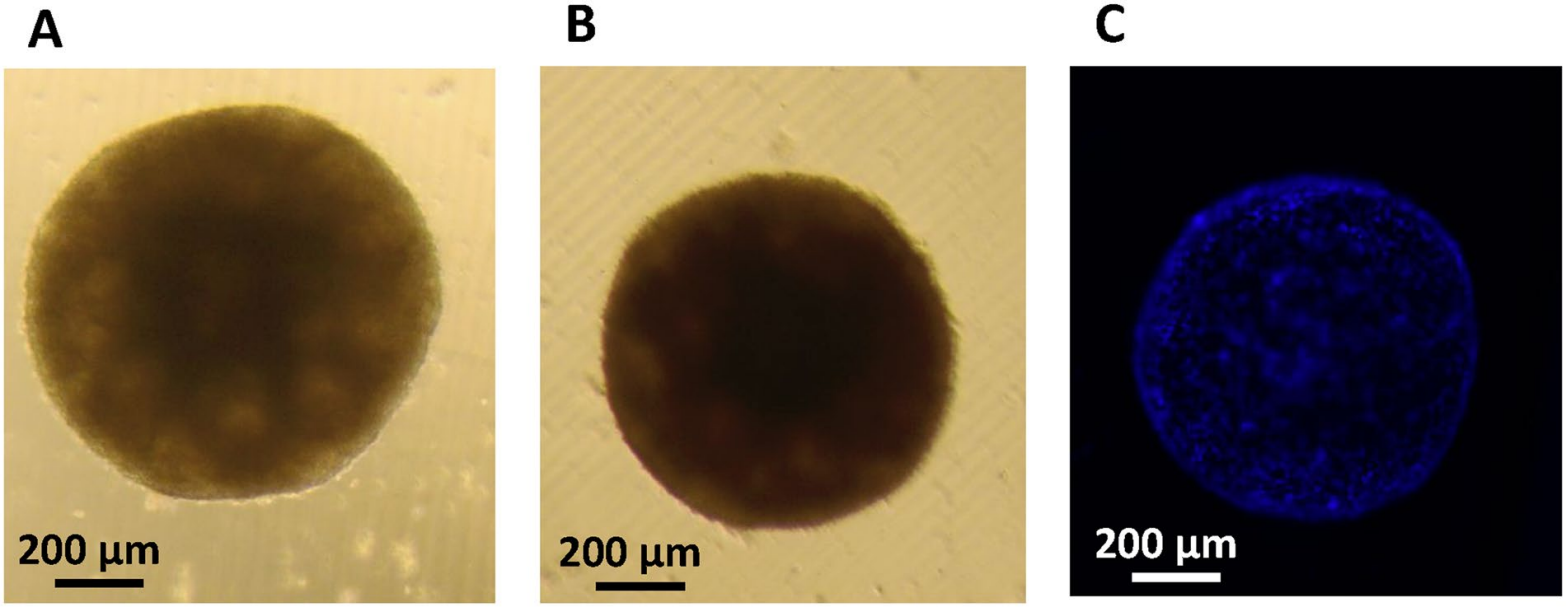
Embryoid body formation. (**A**) Brightfield image of a 21 day-old embryoid body derived from unlabeled human embryonic stem cells (10× magnification). (**B**) An embryoid body 21 days after stem cells were labeled at 0.5 mM MnPNH_2_ for 24 hours and (**C**) corresponding DAPI nuclear stain fluorescence image. Shown in each panel is a single embryoid body formed from thousands of embryonic stem cells; individual cells are seen on the fluorescence image.

Figures 5–7 demonstrate that the contrast agent had no adverse effects on cell colony morphology, cell survival, proliferation, and differentiation potential. In Fig. 5, bright-field microscopy images of representative colonies are shown for unlabeled control cells and cells at 24, 48, and 72 hours after a 24-hour labeling interval. Colonies at 96 hours post-labeling show similar morphology. These images illustrate that other than a slight coloration of cells from MnPNH_2_, the labeling itself had no effect on colony size, shape, and distribution. Cell viability was also unaffected, even for the longest labeling interval of 24 hours (Fig. 6). To further confirm the absence of adverse effects on cell behavior and function, labeled human ESCs were successfully differentiated into embryoid bodies. Every well containing labeled ESCs developed into embryoid bodies, and these all displayed the same size and appearance regardless of their origin (i.e. whether they were differentiated from labeled or unlabeled human ESCs) (Fig. 7).

**Figure 8:**
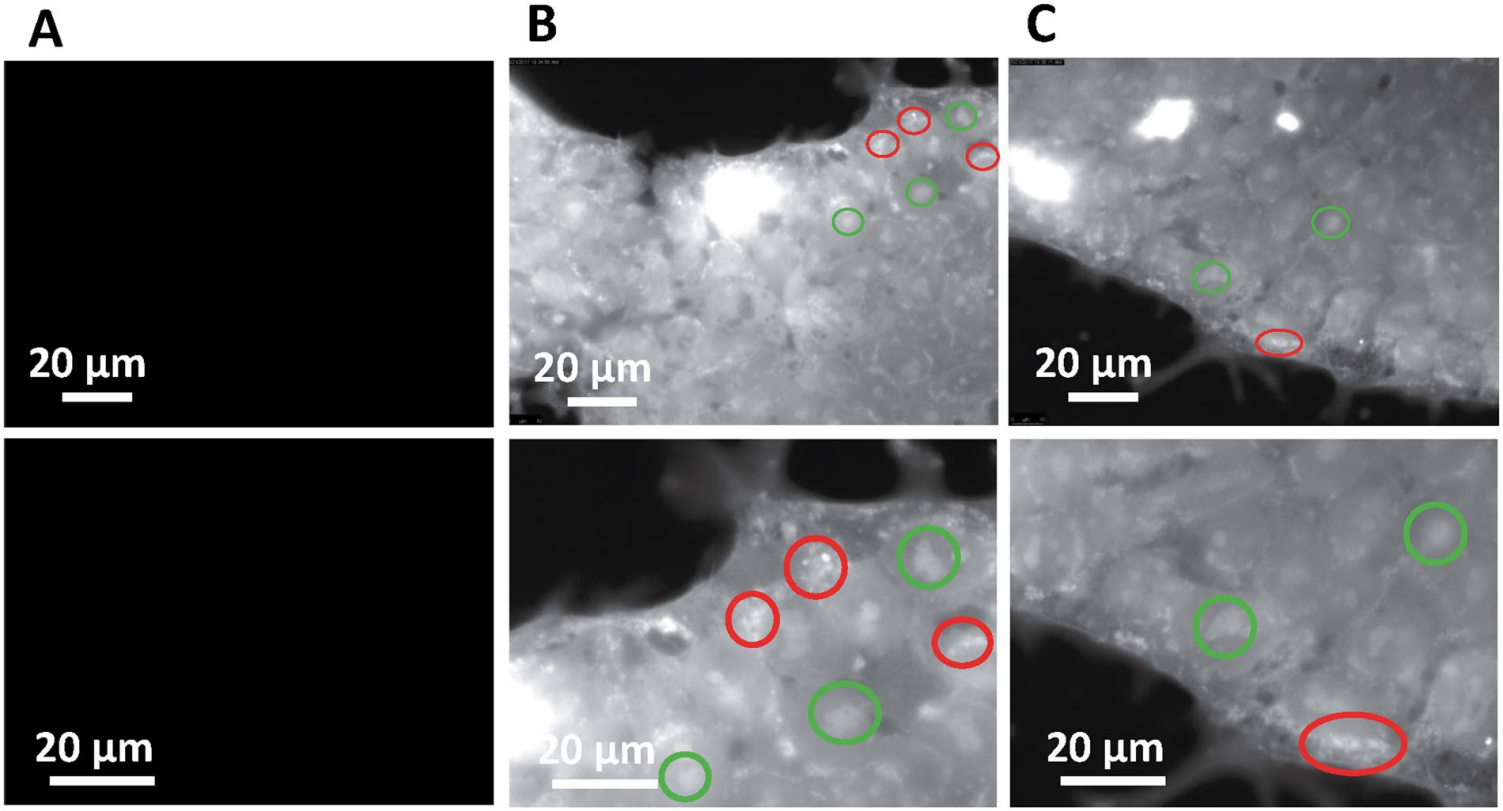
Subcellular distribution of contrast agent. Fluorescence imaging reveals the subcellular distribution of PNH2 after a 24-hour labeling interval. (**A**) Unlabeled cells show no fluorescent signal (63× magnification). (**B**) Cells labeled with 0.5 mM of the apo-porphyrin for 24 hours (63× magnification) with (**C**) further digital zoom. Red circles indicate numerous bright accumulations around the cell periphery and inter-connective space of the colony. Green circles highlight increased signal from higher porphyrin concentration in the nucleus.

Using the non-metalated version of MnPNH_2_ for labeling human ESCs, we were able to map the subcellular distribution of the contrast agent on fluorescent microscopy. Fluorescence was detected throughout the cells, but a stronger signal was observed as clumps near the cell periphery and in the nucleus (Fig. 8). These observations suggest that the contrast agent is, indeed, internalized and not simply bound to edges of the cell membrane, with preferential localization in the nucleus.

**Figure 9:**
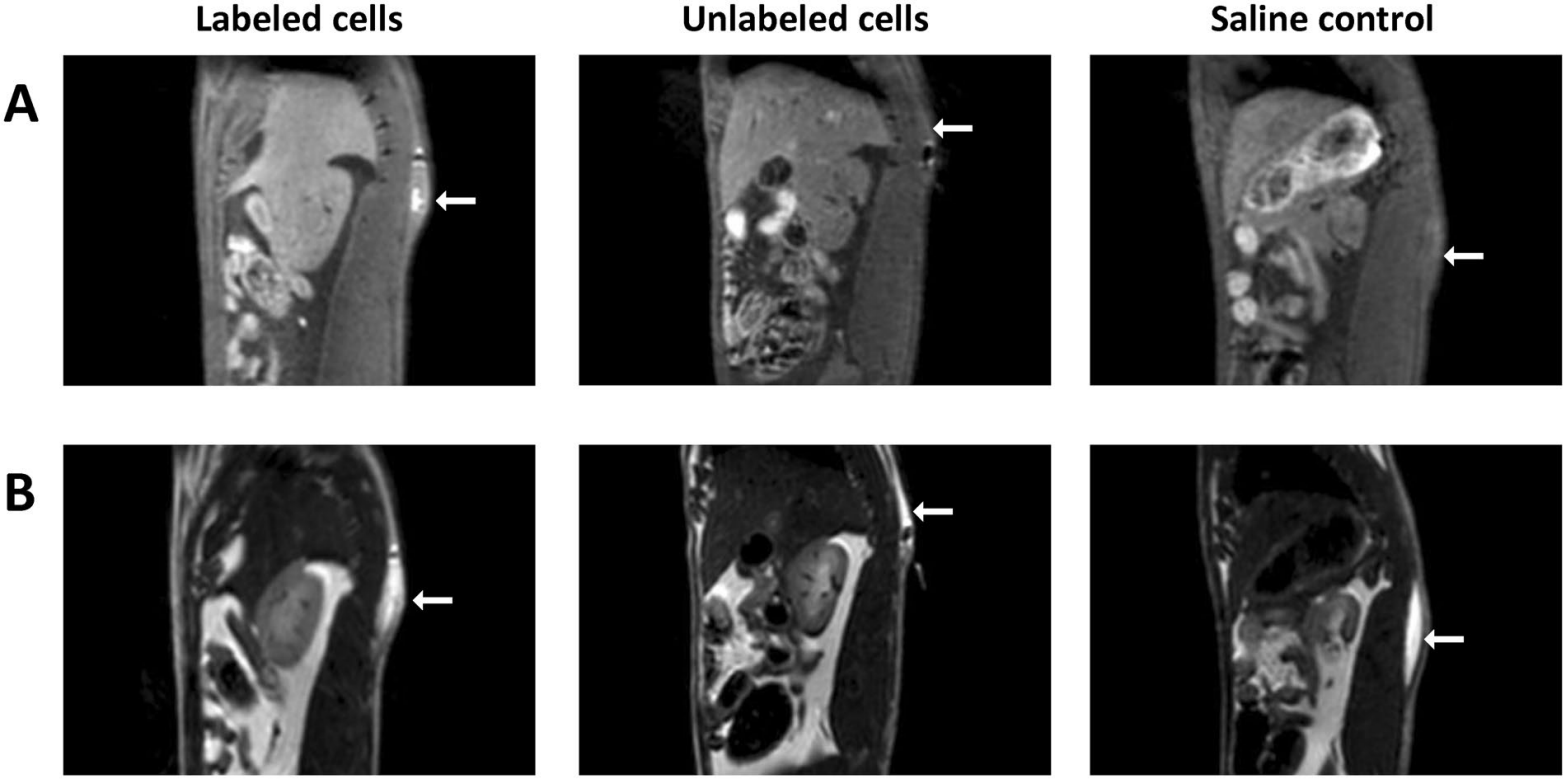
*In-vivo* MR imaging of implanted human embryonic stem cells in rat. Cells labeled at 0.22 mM MnPNH_2_ for 24 hours were injected subcutaneously on the dorsal side of adult rats. T1-weighted spin-echo images with fat suppression (**A**) clearly show an enhancing volume where the labeled cells were injected, whereas unlabeled cells and saline were isointense against native tissue. T2-weighted turbo spin-echo images were acquired to localize the fluid in all injections, independent of whether or not cells were present.

*In-vivo* imaging of rats injected with labeled and unlabeled human ESCs demonstrated superior detection sensitivity afforded by MnPNH_2_ (Fig. 9). Labeled cells can be clearly seen as a brightly enhancing volume at the site of injection on T1-weighted spin-echo images, whereas unlabeled cells and saline are both isointense, as expected. T2-weighted images are shown only to demonstrate their inability to delineate cells, as hyperintensity represents the fluid in which cells are suspended.

## Discussion

The main driving motivation for this work was to create an efficient cell-labeling T1 contrast agent with good potential for clinical translation. This requirement meant that the agent had to be both safe and easily synthesized. We have shown that MnPNH_2_ is easy to synthesize and requires a simple one-step chemical reaction to produce the final functional agent. The single step of metalating the porphyrin can be readily carried out in labs that are not specialized in MR contrast agent chemistry^17^, thus representing a viable alternative for many research groups who are interested in employing MR contrast agents for cell tracking. Another advantage of our agent is that no complicated procedure to assist cell uptake is required (e.g. electroporation), as the agent need only be dissolved in the media in which cells are cultured.

The other major consideration of safety is even more important for any cell-labeling contrast agent intended for meaningful *in-vivo* pre-clinical studies or eventual clinical translation. Meeting this requirement of safety cannot be more critical than in regenerative medicine, where the labeled stem cells are the source for new tissue growth. The labeling agent cannot exert adverse effects on cell function: this includes cell viability, proliferation, and, most importantly for stem cells, differentiation potential. We have shown the safety of MnPNH_2_ on a cellular level through a panel of morphological and functional assessment: light microscopy, trypan blue viability assay, proliferation and embryoid body differentiation^18^. While more extensive testing can be performed, this panel represents the key initial indices that need to be probed to confirm safety.

In regard to T1 efficiency as a cell-labeling contrast agent, MnPNH_2_ produced nearly a two-fold reduction in T1 relaxation times, which enabled high signal-to-noise contrast for labeled cells. The one interesting observation we made was related to the absence of significant T1 changes with incubation concentration when the labeling interval was 2 hours. This implies that the rate of absorption of MnPNH_2_ was too slow and was likely the rate-limiting step for short labeling intervals. To reap the benefit of higher incubation concentrations, a 24-hour labeling interval was necessary. However, notice that changes in T1 did not scale with intracellular Mn content. It is entirely possible that the contrast agent aggregated in the cytoplasm initially, which led to a plateau in T1 despite a higher absolute ion content. With time, aggregation lessened and the agent exerted a greater T1 effect.

To gain an appreciation for MnPNH_2_–induced contrast on MRI, the intracellular distribution of the fluorescent version, PNH_2_, was observed for a labeling interval of 24 hours and an incubation concentration of 0.5 mM. This was done to ensure maximum fluorescence signal. Peak brightness was detected as clumps at the edges of cells, indicating that PNH_2_ aggregated potentially in vacuoles in the cytoplasm. Also, the nucleus appeared brighter than the cytoplasm, indicating overall denser nuclear accumulation, in agreement with a previous study that confirmed nuclear penetration of the apo-porphyrin^19^. Since the agent is present in the nucleus, chromosomal assay or DNA sequencing can be performed in future work to show it is not negatively interacting with the cell’s DNA. Furthermore, real-time microscopy in living cells would give better insight into the dynamics of the agent’s uptake and distribution in cells and possibly shed light on the impact of different labeling intervals.

Further increases in T1 contrast of labeled cells may be desired. To determine the maximum achievable contrast, a much larger range of incubation times and contrast concentrations need to be tested. It would be necessary when testing higher doses to implement more extensive and detailed assays to ensure off-target effects in cells are absent. For example, in addition to the trypan blue exclusion test for cell membrane integrity^20^, we would also perform a TUNEL assay to rule out apoptotic cell death^21^. The potential for genetic or long-term alterations on cell function also need to be ruled out using chromosomal karyotyping, or DNA sequencing, although genetic alternations are not anticipated. Lastly, full differentiation into the desired mature cell type would be performed to ensure both structure and function is maintained.

As with all exogenous contrast agents used for cell labeling, the contrast agent will be diluted as cells divide and the agent becomes distributed amongst daughter cells. Depending on the rate of cell division, one can expect the contrast from labeled cells to be retained over the course of a few days, perhaps even up to a week for slowly dividing cells. In addition to the dilution effect, however, contrast agents will leak back out across the cell membrane unless a complex mechanism is employed to “lock” the agent in^11,22^. Our retention study showed that labeled cells maintained significant contrast within the first 24 hours post-labeling. For many regenerative medicine applications, the first 24 hours post-cell transplantation represents the most challenging interval for cells to survive through. Being able to monitor cell survival and, in moving tissues like the heart, cell retention during this time span is key to informing on whether additional cell injections are needed and where.

Given the preponderance of MR contrast agents utilized for cell tracking, with none approved for clinical use, it is important to compare our agent with other ones that have been employed in the past. Negative contrast iron oxide nanoparticles represent the vast majority of cell-tracking applications. However, they cannot be compared to small molecule T1 agents due to differences in mechanisms of cell uptake and affinity for macrophages. In truth, there is no easily accessible T1 agent for MR cellular imaging. Clinically approved gadolinium-based agents are not designed for labeling cells, as their hydrophilicity renders them impermeable to the cell membrane. Different formulations of T1 agents reported in the literature require specialized chemistry for their synthesis, which render them immediately inaccessible to most labs^22,23^.

A more subtle issue with potential toxicity pertains not to the labeled cells but to the organism receiving the cell transplant. Although manganese is present at low levels physiologically, it is a neurotoxin at high concentrations in the body. Many early studies on neuroimaging have reported toxicity of manganese chloride (MnCl_2_)^24,25^. However, note that these studies used *free* Mn ion, which is very different from the Mn-porphyrin complex we report here. The porphyrin ring provides extremely strong thermodynamic stability of the bound Mn ion, which puts this compound in a league different from MnCl_2_ and Mn nanoparticles. Unlike gadolinium, which is toxic even in minute amounts, Mn is toxic only at high level. In rats, LD50 of MnCl_2_ has been calculated to be 7.5 mmol/kg^23^. If we assume we inject 10 million cells into a 300 g rat (and this is a very high number of cells), each cell carrying the maximum level of Mn we measured at 5.59 × 10^−8^ mmol/cell, we have equivalently 1.86 mmol/kg, which is well below the LD50 level for *free* Mn. So, in the greatly exaggerated scenario where *all* the Mn ions dissociate from the porphyrin ring, we are still administering safe levels of Mn.

Finally, detection sensitivity is the single most important parameter ascribed to any contrast agent, especially for T1 agents that are less sensitively detected than negative-contrast iron oxide nanoparticles. This concept of detection sensitivity goes beyond simply knowing the relaxivity of the agent but considers also the number of cells in a typical imaging voxel, noise level, and so on. In our *in-vitro* studies, there were approximately 75,00 cells per voxel with a volume of 0.5 × 0.5 × 3 mm^3^. A rigorous study of the minimum number of detectable cells is beyond the scope of this manuscript and would require titrating the cell density in cell suspensions *in vitro* and investigating the contrast-to-noise as a function of anatomical location *in vivo*. To answer the question of adequate detection sensitivity for our contrast agent, we took a pragmatic approach and performed an *in-vivo* study in rats. When labeled cells are injected in an animal, cell density is governed naturally by how easily the cells could occupy native tissue, which is very different from the artificial scenario of being densely packed in a cell pellet. Our rat imaging results clearly demonstrate the high detection sensitivity provided by a modest dose of MnPNH_2_ for cell tracking *in vivo*.

There are a number of avenues to explore in future work. One of these is to optimize labeling with respect to contrast agent concentration and labeling interval for human ESCs and to repeat this optimization for several different mature cell types of interest for regeneration. Karyotyping will also be performed now that the key indices of cell function (i.e. viability, proliferation, differentiation) have been shown to be unaffected by labeling. *In-vivo* studies are also planned for preclinical models of stem-cell therapy in relevant anatomical locations for regeneration, such as the heart muscle wall subsequent to a myocardial infarction.

## Conclusions

This study has presented a MRI contrast agent for T1-based cellular imaging and tracking of human embryonic stem cells. We achieved efficient labeling of human ESCs using a manganese-based contrast agent that is simple to synthesize and can be readily adopted in research labs that wish to employ MRI agents for cell labeling. No adverse effects on cell viability or differentiation potential were observed, and subcellular assessment revealed that the agent passed through the cell membrane and even accumulated in the nucleus. Cellular contrast was maintained for 24 hours post-cell labeling, and *in-vivo* imaging of transplanted cells in rats demonstrated superior sensitivity of detection. Future studies will extend this contrast agent to other cell types for tissue regeneration, such as for spinal cord and muscle.

### Data availability

The datasets generated during and/or analysed during the current study are available from the corresponding author on reasonable request.
